# Molecular Patterns Based on Immunogenomic Signatures Stratify the Prognosis of Colon Cancer

**DOI:** 10.3389/fbioe.2022.820092

**Published:** 2022-02-14

**Authors:** Cong Shen, Cong Luo, Zhijie Xu, Qiuju Liang, Yuan Cai, Bi Peng, Yuanliang Yan, Fada Xia

**Affiliations:** ^1^ Department of Thyroid Surgery, Xiangya Hospital, Central South University, Changsha, China; ^2^ National Clinical Research Center for Geriatric Disorders, Xiangya Hospital, Central South University, Changsha, China; ^3^ Department of Urology, Xiangya Hospital, Central South University, Changsha, China; ^4^ Department of Pathology, Xiangya Hospital, Central South University, Changsha, China; ^5^ Department of Pharmacy, Xiangya Hospital, Central South University, Changsha, China

**Keywords:** immune, colon cancer, signature, risk score, prognosis

## Abstract

**Background:** Colon cancer is an aggressive and heterogeneous disease associated with high morbidity and mortality. The immune system is intimately involved in tumorigenesis and can influence malignant properties at the protein, epigenetic, and even genomic levels by shaping the tumor immune microenvironment (TIM). However, immune-related molecules that can effectively predict the prognosis of colon cancer remain under exploration.

**Methods:** A total of 606 patients from TCGA and GEO databases were employed in our study, in which 429 cases were set as the training cohort and 177 were defined as the validation cohort. The immune infiltration was evaluated by ESTIMATE, TIMER, and CIBERSORT algorithms. The risk signature was constructed by LASSO Cox regression analysis. A nomogram model was generated subsequent to the multivariate Cox proportional hazards analysis to predict 1-, 3-, and 5-year survival of patients with colon cancer.

**Results:** Infiltrating immune cell profiling identified two colon cancer clusters (Immunity_L group and Immunity_H group). The abundances of immune cells were higher in the Immunity_H group, which indicated a better prognosis. Through further statistical analysis, we identified four genes which were highly correlated with prognosis and representative of this gene set, namely *ARL4C*, *SERPINE1*, *BST2*, and *AXIN2.* When the patients were divided into low- and high-risk groups based on their risk scores, we found that patients in the high-risk group had shorter overall survival time. Moreover, a nomogram including clinicopathologic features and the established risk signature could robustly predict 1-, 3-, and 5-year survival in patients with colon cancer.

**Conclusion:** We identified two distinct immune patterns by analyzing clinical and transcriptomic information from colon cancer patients. A subsequently constructed immune-related gene-based prognostic model as well as a nomogram model can be used to predict the prognosis of colon cancer, thereby guiding risk stratification and treatment regimen development for colon patients.

## Introduction

Recent cancer statistics reveals a high incidence of 10.2% for human colon cancer, while the mortality rate is up to 9.2%, rising from the fourth to the second place in the oncological field, seriously threatening human life health (Bray et al., 2018; Siegel et al., 2020a). Surgical removal of cancerous tissue, combined with radiotherapy and chemotherapy (if necessary), has been the mainstay of combating colon malignancies (Nie et al., 2020; Liao et al., 2021). Due to advances in systemic drug targeting and surgical techniques, the prognosis of colon cancer patients will be significantly improved if they are diagnosed at an early stage (Labianca et al., 2010). Therefore, accurate grading/staging of colon cancer is helpful for the development of treatment options as well as the prognosis of patients.

Current prognostic prediction mainly relies on the Tumor, Nodes, Metastases (TNM) classification system (clinical level) and histopathological criteria (histological level) (Pagès et al., 2018). Unfortunately, patients still demonstrated an unpleasing survival outcome due to the recurrence, metastasis, and resistance to the agents. Clinicians and researchers have been searching for novel treatment strategies in the hope for achieving better results, and they focused on the cellular and molecular levels to identify valuable markers and tumor-cell differentiation events. A growing number of studies have demonstrated the role of gene mutation status, gene expression levels, and signaling pathway alterations in tumor initiation and progression, but accurately identifying prognostic factors that can provide targets for therapy remains difficult at the genomic level (Beane et al., 2009; Nagy et al., 2018). The immune system is intimately involved in tumorigenesis and can influence malignant properties at the protein, epigenetic, and even genomic levels by shaping the tumor immune microenvironment (TIM) (Deng et al., 2021). Recently, a large body of evidence has shown that immune-related molecules are of great value in predicting prognosis and assessing therapeutic efficacy (Binnewies et al., 2018).

The TIM is a complex system composed of multiple immune cells infiltrating into tumor tissue and various cytokines and chemokines secreted by them (Mallmann-Gottschalk et al., 2019). In there, natural killer (NK) cells can secrete cytokines, such as interferon (IFN)-gamma and tumor necrosis factor (TNF)-α, to exert an immunosuppressive phenotype *via* inhibiting tumor cell proliferation and tumor angiogenesis (Liu et al., 2018). Moreover, tumor associated macrophages (TAMs) and regulatory T cells (Tregs) mediate a suppressed tumor microenvironment which helps tumor cells achieve immune escape, and promotes the development of malignancy (Pan et al., 2020).

To address these suppressive immune phenotypes, targeting immune-tumor cell interactions has become more intensively studied, and immunotherapy has emerged as a promising area of cancer treatment and has demonstrated its impressive clinical value in colon cancer. This is mostly attributed to the face of immune checkpoint inhibitors as antitumor agents, such as programmed death 1 receptor (PD1) and cytotoxic T lymphocyte antigen 4 (CTLA-4) inhibitors (Yaghoubi et al., 2019). However, immunotherapy for colon cancer is still imperfect, for example, the effect of immunotherapy cannot be evaluated in advance. In conclusion, immunogenomic classification will help to guide the identification and effective treatment of early colon cancer and improve the accuracy of prognosis evaluation.

In this study, we performed an immunogenomic profiling of patients with colon cancer and divided them into two distinct subtypes: high immunity (Immunity_H) and low immunity (Immunity_L). We focused on analyzing two independent cohorts of colon cancer patients to identify genes highly associated with prognosis; based on their expression levels, the patients were allocated risk scores. We then combined clinicopathological characteristics and the risk scores to establish a model to accurately predict the survival rate of patients with colon cancer. This analysis is of great significance for the survival prediction of patients with colon cancer and provides a potential target for its treatment.

## Materials and methods

### Data Source and Extraction

The data we used for analysis were obtained from The Cancer Genome Atlas (TCGA, https://portal.gdc.cancer.gov/) (The Cancer Genome Atlas Network, 2012). In the dataset, 429 colon adenocarcinoma (COAD) patients had complete clinicopathological and transcriptomic expression data. Thus, they were enrolled as the training set. In addition, we also downloaded three datasets, including GSE17536 (*n* = 177), GSE17537 (*n* = 55), and GSE103479 (*n* = 156) in the Gene Expression Omnibus (GEO, https:/www.ncbi.nlm.nih.gov/geo/) to validate the results (Smith et al., 2010; Dienstmann et al., 2019). The crude RNA expression data Fragments Per Kilobase of exon model per Million mapped fragments (FPKM) were transformed into Transcripts Per Kilobase of exon model per Million mapped reads (TPM) for a better statistical evaluation.

### Clustering for Distinct Immune Patterns in Colon Cancer

We screened and quantified 28 immune cell types in the TCGA-COAD dataset using the single sample gene set enrichment analysis (ssGSEA) algorithm (Barbie et al., 2009). For each independent dataset, an enrichment score was calculated to represent the enrichment level of 28 immune cell types for each tumor sample. Based on these ssGSEA scores, we performed consensus clustering on TCGA-COAD. In brief, cluster analysis was performed using “ConsensusClusterPlus” (Wilkerson and Hayes, 2010), using agglomerative k-means clustering with a 1-Pearson correlation distance and resampling 80% of the samples for 10 repetitions. The optimal number of clusters was determined using the empirical cumulative distribution function plot. A principal component analysis (PCA) was conducted to analyze the distinguishing ability of the clustering.

### Quantification of Immunotherapy Response, Major Histocompatibility Complex, and Immune Microenvironment

We used the “ESTIMATE” package to calculate the Immune, Stromal, and ESTIMATE scores for each sample to show the component fractions and tumor purity (Yoshihara et al., 2013). ImmuCellAI (Immune Cell Abundance Identifier) is a tool to estimate the abundance of 24 immune cells from gene expression dataset including RNA-Seq and microarray data (Miao et al., 2020). We applied it to predict patients’ response to immune checkpoint blockade therapy. The gene expression of the major histocompatibility complex (MHC), human leukocyte antigen (HLA), has also been explored in different clusters. Moreover, the abundances of diverse immune and stromal components were calculated using the “IOBR” R package (https://github.com/IOBR/IOBR), which is designed for multi-omics immuno-oncology biological research to decode tumor microenvironment and signatures (Zeng et al., 2021). Specifically, five algorithms built into the tool, including CIBERSORT (Newman et al., 2015), immunophenoscore (IPS) (Charoentong et al., 2017), MCP-counter (Becht et al., 2016), xCell (Aran et al., 2017), and EPIC (Racle et al., 2017), were used to calculate the scores of 51 infiltrating (immune and stromal) cells in each sample.

### Profiling of the Differentially Expressed Prognostic Genes Related to Colon Cancer Subtype-specific Immunity

We performed difference analysis between the Immunity_Low (Immunity_L) group and Immunity_High (Immunity_H) group using the “limma” package (Ritchie et al., 2015). The absolute values of differential expression multiples >1.5 and p < 0.05 were used as the criteria for screening differentially expressed genes. The up- and down-regulated immune related genes (IRGs) in colon cancer were displayed in volcano plot. For functional analysis, we used the “org.Hs.eg.db” package (version 3.1.0) to perform the gene ontology (GO; including biological process, molecular function, and cellular component) and Kyoto Encyclopedia of Genes and Genomes (KEGG) pathway annotation. A Benjamini–Hochberg false discovery rate (BH-FDR) <0.05 was considered statistically significant. The enrichment results were displayed as histogram and lollipop charts.

### Identification of Prognostic Genes and Construction of Immune Signature

Univariate Cox regression analysis based on differentially expressed genes was used to screen immune related genes significantly related to the prognosis of colon cancer with p < 0.05 as the threshold. Subsequently, a Least Absolute Shrinkage and Selection Operator (LASSO) Cox regression analysis with “glmnet” package (Tibshirani, 1996) was used to further identify essential genes and allocate coefficients for them. The risk score of each sample was calculated using the following formula:

$$\mathrm{Risk score}=\sum_{i=1}^{n}Coefi*xi$$

where *Coef*_*i*_ is the risk coefficient of each factor and *x*_*i*_ is the mRNA expression value (logarithmic transformed TPM) of each factor. After determining the optimal cut-off value of risk score through “survival” and “survminer” packages, patients were divided into *low risk* and *high risk* groups correspondingly. Survival curves were used to show the differences in survival time and survival probability between *high risk* and *low risk* patients based on the Kaplan–Meier method. The area under curve (AUC) of receiver operating characteristic (ROC) curves represented the predictive accuracy (Heagerty et al., 2000). Univariate and multivariate Cox regression models were used to analyze whether the risk score was able to independently predict survival in patients with colon cancer.

### Establishment of Nomogram Prognosis Prediction Model

Nomograms are widely used to predict the prognosis of the disease, so we drew a nomogram based on all independent prognostic factors identified by multivariate Cox regression analysis to predict the survival probability of patients within 1, 3, and 5 years by using the “rms” package. For practical application, we created a dynamic nomogram through “DynNom” package and built an interactive web-based tool with Shiny (https://shiny.rstudio.com/) (Supplementary Material S1). A nomogram calibration curve was plotted to judge nomogram accuracy by observing the relationship between predicted probability and actual incidence. The practicability of 1-, 3-, and 5-years OS was evaluated by ROC curves. The prognostic ability of the nomogram and other predictors (risk score, N stage, and M stage) for survival were evaluated by decision curve analysis (DCA) curves using the “rmda” R package.

### Cell Culture

Normal human colon epithelial cells (NCM-460) and two human colon cancer cell lines (HCT116, HCT8) were obtained from the cell bank at the Chinese Academy of Sciences (Shanghai, China). All cells were authenticated by short tandem repeat (STR) profiling upon receipt and were propagated for less than 6 months after resuscitation. All cell lines were cultured in Dulbecco’s modified eagle medium (DMEM) medium with 10% fetal bovine serum (FBS; Thermo Fisher Scientific, Waltham, MA, USA). These cell lines were maintained in a humidified chamber containing 5% CO_2_ at 37°C.

### RNA Extraction and qPCR

Total RNA from cultured cells and fresh tissues was extracted with Trizol regent (Thermo Fisher Scientific). We used NanoDrop and an Agilent 2,100 bioanalyzer (Thermo Fisher Scientific) to determine the concentration of extracted total RNA. cDNA was obtained by reverse transcription using a reverse transcription kit (Hiscrip II Q RT SuperMix for qPCR; Vazyme, Nanjing, China) according to the manufacturer’s protocol. Quantitative real-time polymerase chain reaction amplification was performed with SYBR Green PCR master mix (Takara, Japan) according to the manufacturer’s protocol. Primers were designed as follows: SERPINE1, forward, 5′-AGT​GGA​CTT​TTC​AGA​GGT​GGA-3′, reverse, 5′-GCC​GTT​GAA​GTA​GAG​GGC​ATT-3′; ARL4C, forward, 5′-CCA​GTC​CCT​GCA​TAT​CGT​CAT-3′, reverse, 5′-TTC​ACG​AAC​TCG​TTG​AAC​TTG​A-3′; BST2, forward, 5′-CAC​ACT​GTG​ATG​GCC​CTA​ATG-3′, reverse, 5′-GTC​CGC​GAT​TCT​CAC​GCT​T-3′; AXIN2, forward, 5′-TAC​ACT​CCT​TAT​TGG​GCG​ATC​A-3′, reverse, 5′-TTG​GCT​ACT​CGT​AAA​GTT​TTG​GT-3′. GAPDH was used as an endogenous control, and relative gene expression was determined by the comparative 2^−ΔΔCT^ method.

### Immunohistochemistry

The Human Protein Atlas (HPA) (https://www.proteinatlas.org/) is a program for mapping human proteins in cells, tissues, and organs using integration of various omics technologies (Uhlen et al., 2017; Sun et al., 2018). We obtained representative immunohistochemistry results of the four target proteins in colon cancer and normal colon tissues from the tissue atlas and pathology atlas in HPA database, respectively.

### Statistical Analysis

**FIGURE 1:**
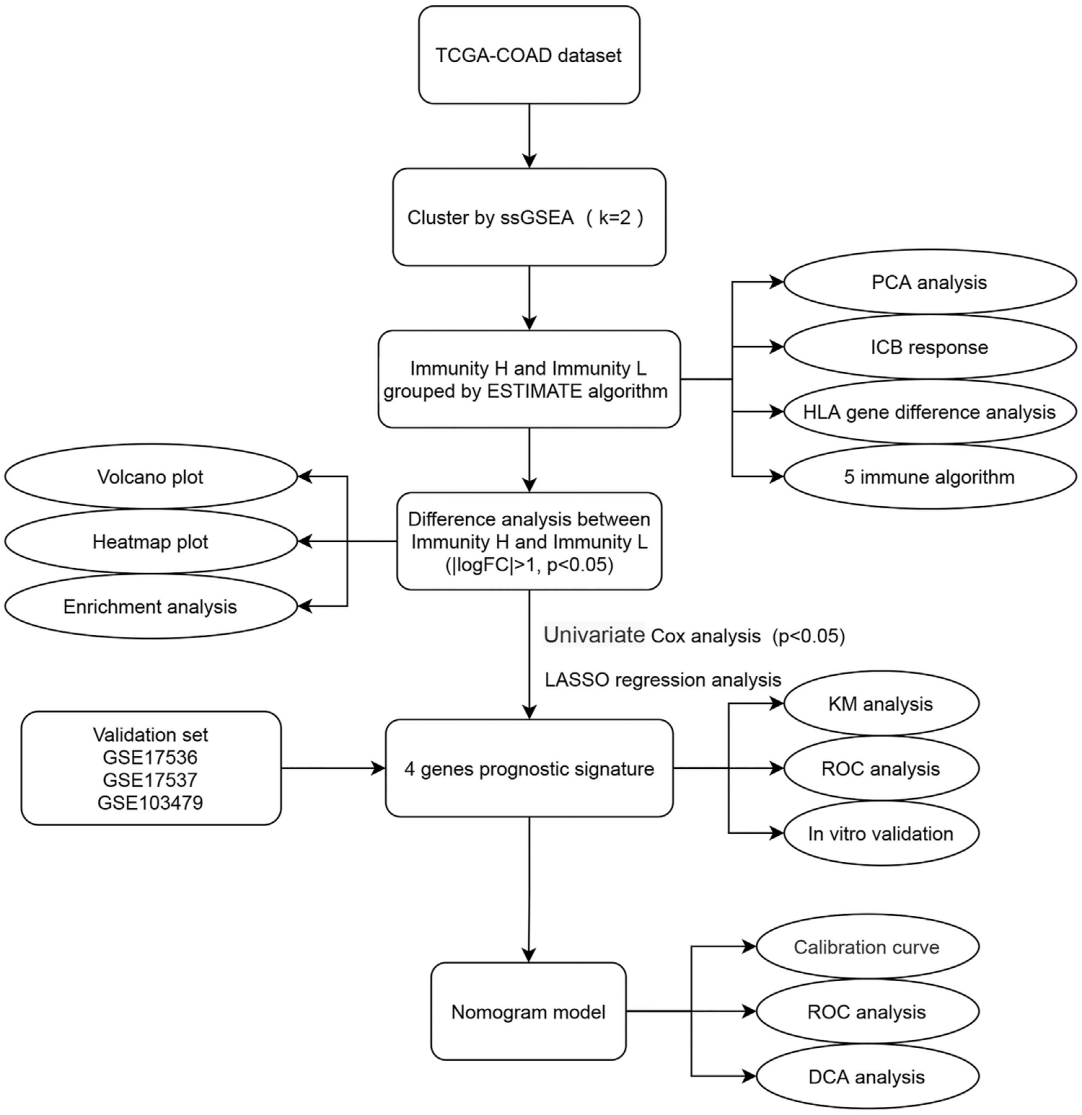
Flow chart of this study. The square element represents the research process, and the oval element represents the specific research method and result.

In this study, Kaplan–Meier method was used to estimate the overall survival rate of different groups, and log-rank was used to test the significance. The inter-group comparisons were achieved by using Wilcoxon rank sum test. The chi-square test was used to compare the clinicopathologic features (age, gender, TNM stage, and AJCC stage) between the *low risk* group and *high risk* group. Univariate and multivariate Cox regression analyses were utilized to evaluate the independent prognostic value of the risk signature regarding OS. Statistical analyses were made using R software (version 4.0.3). Most visualizations were achieved by “ggplot2” package. In most situations, p < 0.05 was used as a significant threshold if not otherwise specified Figure 1.

## Results

### Infiltrating Immune Cell Profiling Identified Two Colon Cancer Clusters

**FIGURE 2:**
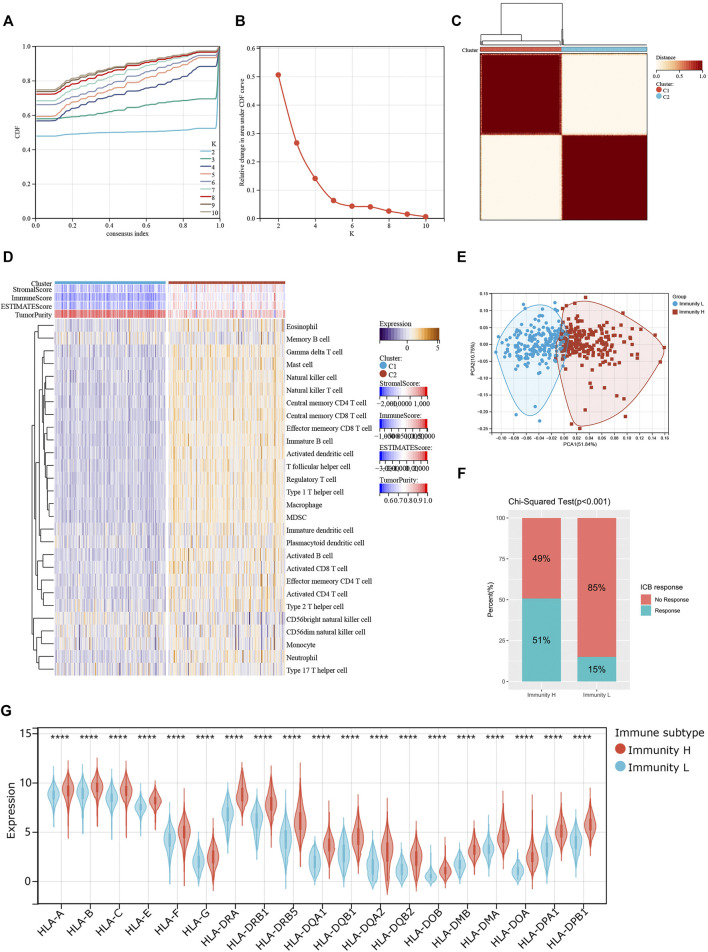
Two patterns of colon cancer based on immune cell clustering. **(A)** Cumulative distribution function (CDF) under different clustering numbers. **(B)** Relative change in area under CDF curve with different clustering numbers. **(C)** Clustering heatmap when *k* = 2. **(D)** Heatmap showing the differences in immune cell ssGSEA scores between two clusters. **(E)** PCA regarding two clusters. **(F)** The differences in response rates to immune checkpoint blockades between two clusters. **(G)** The expressions of HLA genes between two clusters.

We screened and analyzed 28 immune cell types by ssGSEA for every tumor sample (Supplementary Table S2). Then, we performed consensus clustering on TCGA-COAD dataset based on the ssGSEA scores, which represented the activity or infiltration levels of immune cells in the tumor sample. Finally, the patients were divided into two unique immune clusters (C1 and C2; Figures 2A–C). We found that the ssGSEA scores of all the immune cell types were higher in C2 than those in C1 (Figure 2D). The C1 and C2 were thus defined as the Immunity_Low (Immunity_L) group and Immunity_High (Immunity_H) group, respectively (Supplementary Table S3). Furthermore, the heatmap clearly demonstrated that the Immunity_H group possessed higher stromal scores, immune scores, and ESTIMATE scores when compared with the Immunity_L group (Figure 2D). Correspondingly, the Immunity_H group exhibited lower tumor purity.

PCA result further confirmed the reliability of this clustering, as the subgroups could be significantly distinguished (Figure 2E). More importantly, the Immunity_H group showed a higher response rate to immune checkpoint blockers than the Immunity_L group (51 vs. 15%; p < 0.001; Figure 2F). At the same time, we found that most HLA genes were expressed at higher levels in the Immunity_H group than in the Immunity_L group (Figure 2G). These results collaborated that our clustering significantly distinguished colon cancers into two groups with distinct immune landscapes.

**FIGURE 3:**
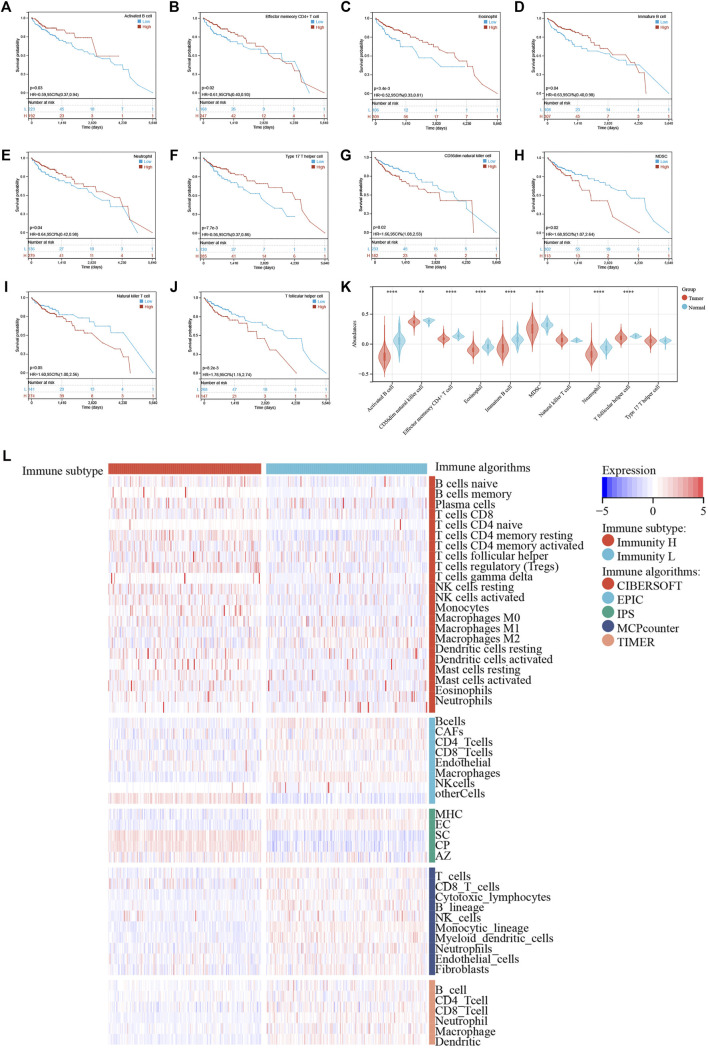
**(A–J)** Kaplan–Meier survival curves of individual cell types with significant prognostic values. **(K)** Differences in abundance of 10 immune cells between normal and tumor tissues. **(L)** Immune cell abundances estimated by five algorithms (CIBERSORT, EPIC, IPS, MCPcounter, and TIMER) between two clusters.

Next, we examined the relationship between individual immune cell ssGSEA score and COAD patient’s overall survival and found that 10/28 cell types were significantly correlated with patient prognosis. Specifically, activated B cell (p = 0.03; Figure 3A), effector memory CD4^+^ T cell (p = 0.02; Figure 3B), eosinophil (p = 3.4e-3; Figure 3C), immature B cell (p = 0.04; Figure 3D), neutrophil (p = 0.04; Figure 3E), and type 17 T helper cell (Th17; p = 7.7e-3; Figure 3F) were beneficial to patient survival, as a patient with higher scores of these cell types would have a higher survival probability, whereas CD56dim NK cell (p = 0.02; Figure 3G), myeloid-derived suppressor cell (MDSC; p = 0.02; Figure 3H), natural killer T cell (p = 0.05; Figure 3I), and T follicular helper cell (Tfh; p = 8.2e-3; Figure 3J) were unfavorable to patient survival, as a patient with higher scores of these cell types would have a worse survival. We then compared the abundances of these immune cells between normal and tumor tissues. Except for the natural killer T cell and type 17 T helper cell, the remaining eight immune cells had generally lower abundance in tumor tissues than in normal tissues (Figure 3K). We used five algorithms to demonstrate differences in the infiltrating cells between the two groups. Overall, the abundances of immune cells were higher in the Immunity_H group, especially for those calculated by CIBERSOFT algorithm (Figure 3L).

### Differentially Expressed Genes Related to Colon Cancer Cluster-Specific Immunity Were Identified and Verified

**FIGURE 4:**
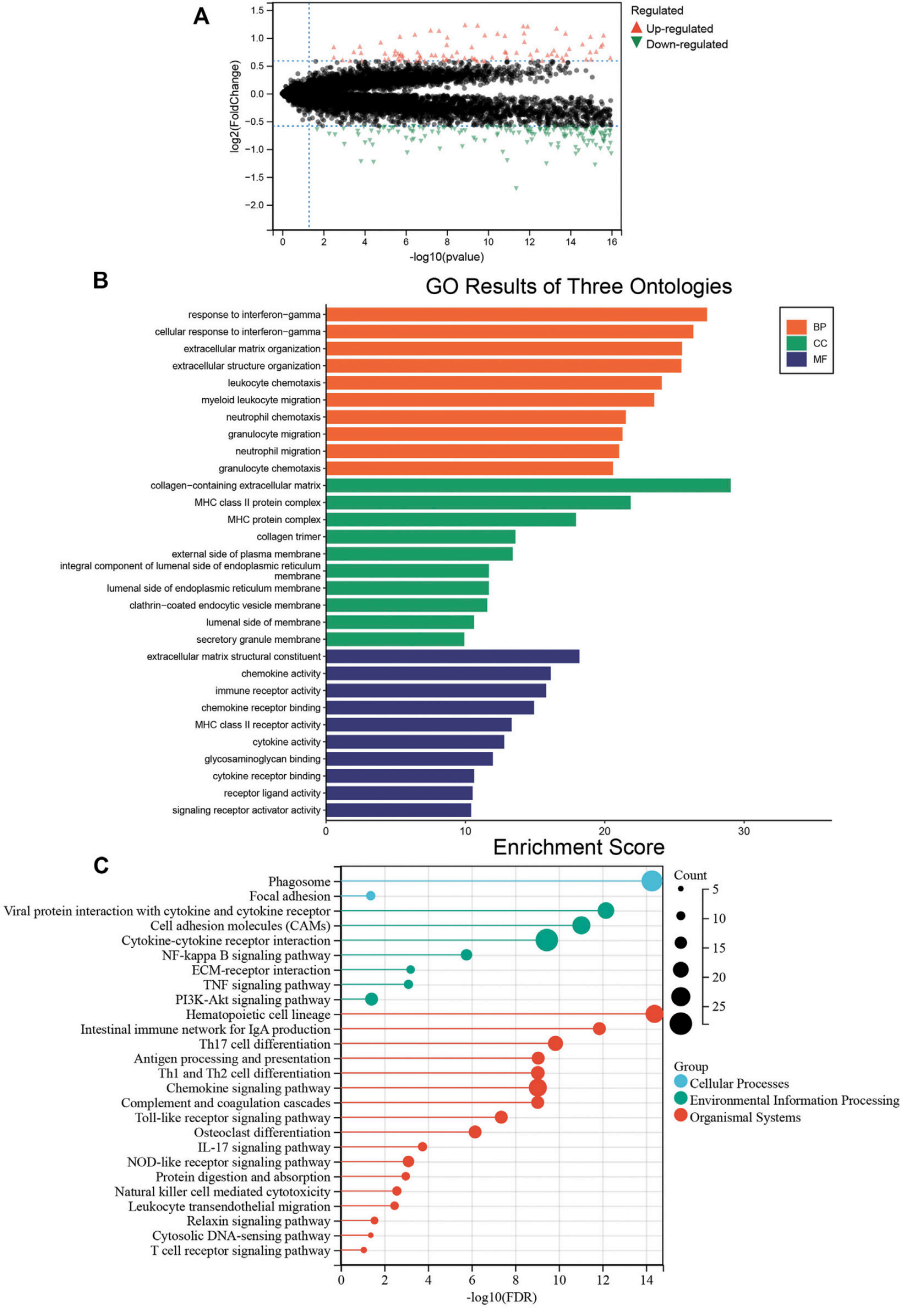
The biological functions of differentially expressed genes between two clusters. **(A)** The differentially expressed genes between the Immunity_L and Immunity_H groups. Red triangles represent up-regulated genes and green triangles represent down-regulated genes. **(B)** Enrichment results of GO terms. Orange: biological process, green: cellular component, purple: molecular function. **(C)** Enrichment results of KEGG pathways. Blue: cellular process, green: environmental information processing, red: organismal systems.

We analyzed the differentially expressed genes between the two colon cancer immune subtypes in TCGA-COAD cohort. A total of 18 genes were up-regulated in the immunty_H group and down-regulated in the Immunity_L group. Conversely, 259 genes were down-regulated in the immunty_H group and up-regulated in the Immunity_L group (Supplementary Table S4; Figure 4A). Through GO enrichment analysis, we found these differentially expressed genes were mostly located on collagen-containing extracellular matrix, involved in biological processes including response to interferon-gamma and extracellular matrix organization. In terms of molecular functions, these genes were mainly involved in extracellular matrix structural constituent and chemokine activity (Figure 4B). The results were consistent to the well-established immune processes. Furthermore, KEGG enrichment analysis concluded that these genes mainly participated in the cellular processes of phagosome, environmental information processing such as cytokine-cytokine receptor interaction. Besides, they were enriched in the organismal systems including hematopoietic cell lineage, Th17 cell differentiation, and chemokine signaling pathway (Figure 4C). These results largely indicated that these differentially expressed genes derived from immunogenomic clusters were closely linked to immune-related pathways.

### A Four-Gene Risk Signature was Constructed in the Training Set

**FIGURE 5:**
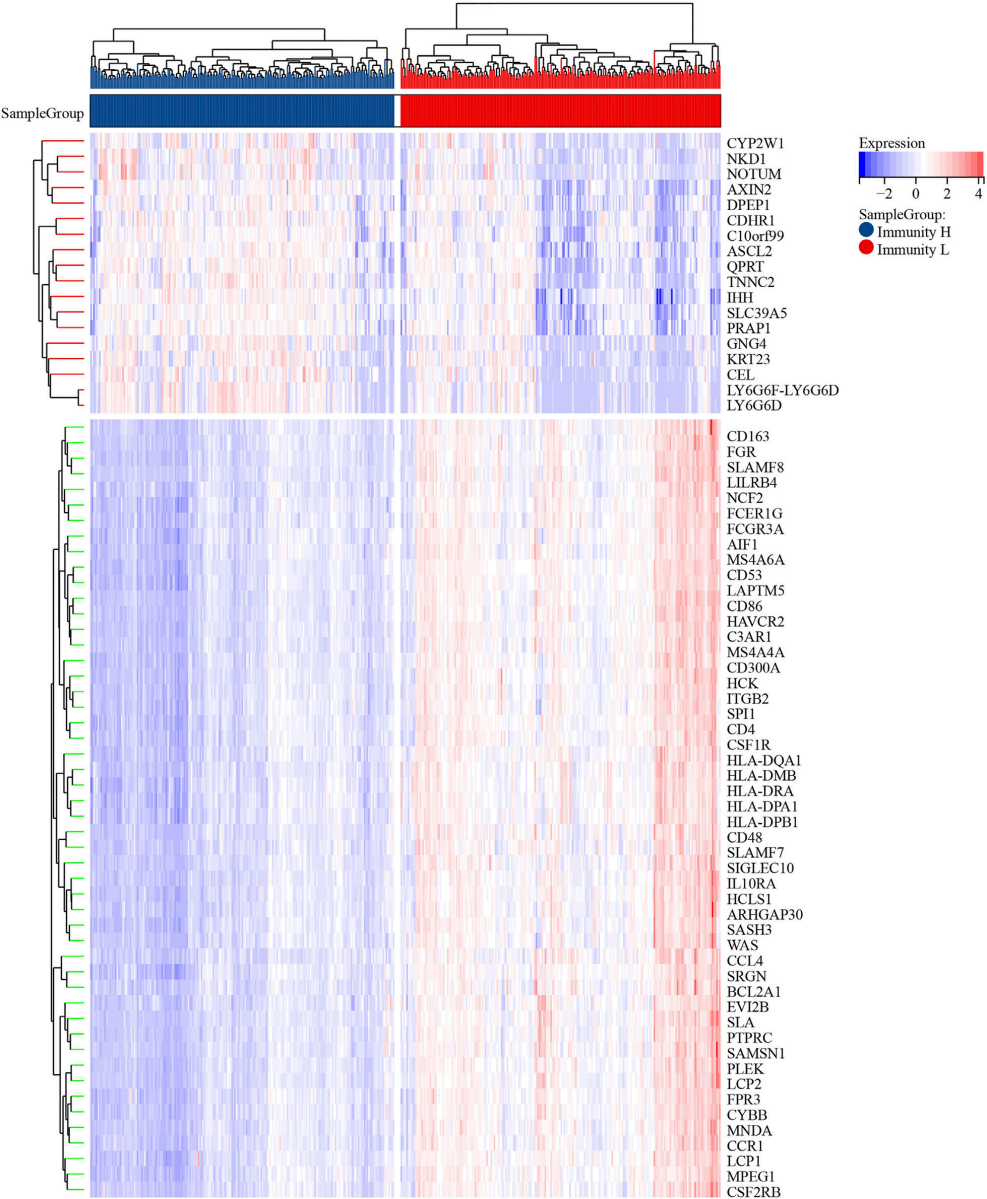
The heatmap of differentially expressed genes.

**FIGURE 6:**
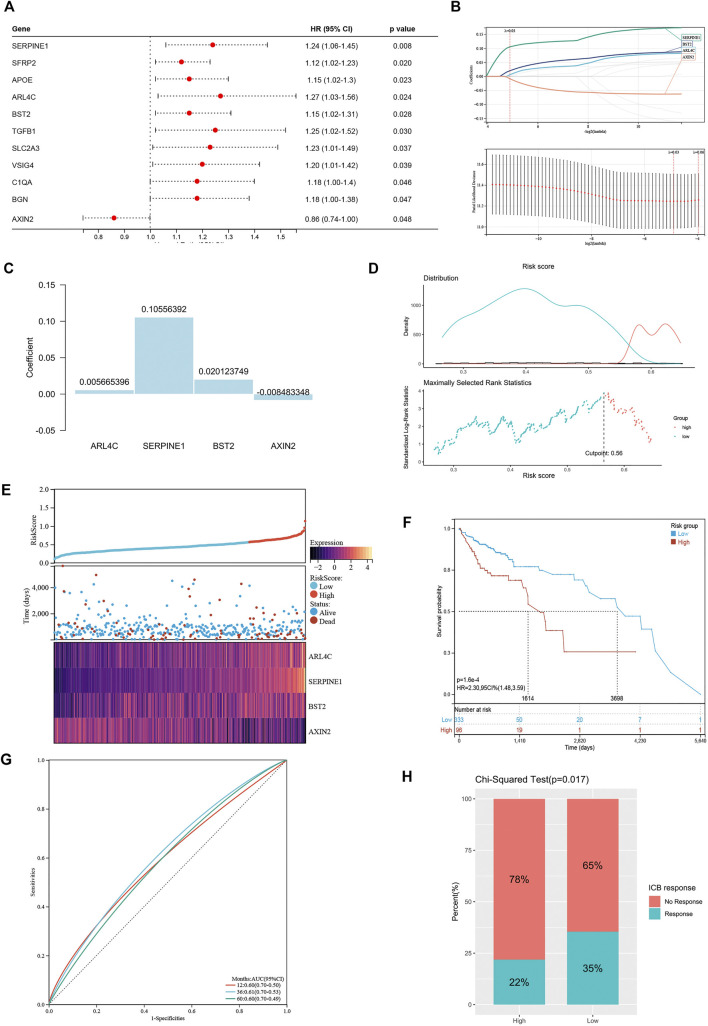
Construction of risk signature based on differentially expressed genes. **(A)** The forest plot of differentially expressed genes with prognostic significance. **(B)** The screening of coefficients and likelihood deviance under LASSO analysis. **(C)** The coefficients of model genes. **(D)** Best cutoff value selected by the log-rank test. **(E)** The relationship between risk score, survival status, and model gene expression. **(F)** The Kaplan–Meier survival curve showing the difference between *high risk* and *low risk* groups in TCGA-COAD. **(G)** The ROC curves regarding 1-, 3-, and 5-year survival outcomes in TCGA-COAD. **(H)** The differences in response rates to immune checkpoint blockades between two groups.

These differentially expressed genes showed a completely distinct expression patterns between the immunty_H group and the Immunity_L group (Figure 5). By univariate Cox regression analysis, we screened 11 differentially expressed genes that were significantly associated with prognosis (Figure 6A), of which *AXIN2* was a protective factor because it harbored a hazard ratio (HR) of less than 1 (HR = 0.861, p = 0.0481), while the remaining 10 genes were risk factors: *SERPINE1* (HR = 1.24, p = 0.0076), SFRP2 (HR = 1.12, p = 0.0203), APOE (HR = 1.15, p = 0.0230), *ARL4C* (HR = 1.27, p = 0.0236), *BST2* (HR = 1.15, p = 0.0283), TGFB1 (HR = 1.25, p = 0.0302), SLC2A3 (HR = 1.23, p = 0.0369), VSIG4 (HR = 1.2, p = 0.0389), C1QA (HR = 1.18, p = 0.0455), and BGN (HR = 1.18, p = 0.0466). We performed further screening of these genes by LASSO regression analysis and identified four genes which were highly correlated with prognosis and representative of this gene set, namely *ARL4C*, *SERPINE1*, *BST2*, and *AXIN2* (Figure 6B).

The LASSO analysis also allocated coefficients for these four genes (Figure 6C), thus facilitating the assignment of risk score to each patient. Under the best cut-off value of 0.56 detected by log-rank test (Figure 6D), we displayed risk scores and survival status distributions as Figure 6E. The *high risk* group had more mortality events. And it clearly showed that *ARL4C*, *SERPINE1*, and *BST2* expression increased with increasing risk score, whereas the expression of the *AXIN2* decreased with elevating risk score. The clinicopathological characteristics between the high and low risk groups are summarized in Table 1. It shows that the risk signature was an independent indicator of prognosis as the characteristics were not significantly different between the two groups, including age (p = 0.097), gender (p = 0.36), AJCC pathologic stage (p = 0.22), T stage (p = 0.052), N stage (p = 0.46), M stage (p = 0.48), and disease type (p = 0.40). Kaplan-Meier survival curves depicted that colon cancer patients with higher risk scores significantly had worse clinical outcomes (HR = 2.17, 95% CI 1.41–3.35, p < 0.001; Figure 6F). The ROC curves demonstrated that the risk signature harbored a promising ability to predict OS in the TCGA-COAD cohort (AUC: 1 year = 0.60, 3 years = 0.61, 5 years = 0.60; Figure 6G). Besides, we also analyzed the response to immune checkpoint blockers in the *high risk* and *low risk* groups. The result showed that the *low risk* group showed higher response rate to immune checkpoint blockers than the *high risk* group (35 vs. 22%; p = 0.017; Figure 6H).

**TABLE 1:** Clinicopathological characteristics between the *low risk* and *high risk* groups

Characteristics	Total (N = 429)	*Low risk* (N = 333)	*High risk* (N = 96)	p Value^a^
Age				0.59
Mean ± SD	66.70 ± 12.77	66.52 ± 12.75	67.31 ± 12.88	
Median [min-max]	69.00 [31.00,90.00]	68.00 [31.00,90.00]	69.00 [34.00,89.00]	
Gender				0.87
Female	202 (47.09%)	158 (36.83%)	44 (10.26%)	
Male	227 (52.91%)	175 (40.79%)	52 (12.12%)	
AJCC stage				<0.01
Stage I	74 (17.25%)	68 (15.85%)	6 (1.40%)	
Stage II	170 (39.63%)	131 (30.54%)	39 (9.09%)	
Stage III	123 (28.67%)	89 (20.75%)	34 (7.93%)	
Stage IV	62 (14.45%)	45 (10.49%)	17 (3.96%)	
T stage				<0.01
T1	9 (2.10%)	9 (2.10%)	0 (0.0e + 0%)	
T2	75 (17.48%)	69 (16.08%)	6 (1.40%)	
T3	297 (69.23%)	221 (51.52%)	76 (17.72%)	
T4	48 (11.19%)	34 (7.93%)	14 (3.26%)	
N stage				0.01
N0	253 (58.97%)	208 (48.48%)	45 (10.49%)	
N1	99 (23.08%)	73 (17.02%)	26 (6.06%)	
N2	77 (17.95%)	52 (12.12%)	25 (5.83%)	
M stage				0.39
M0	367 (85.55%)	288 (67.13%)	79 (18.41%)	
M1	62 (14.45%)	45 (10.49%)	17 (3.96%)	
Disease type				0.35
Adenocarcinoma	372 (86.71%)	292 (68.07%)	80 (18.65%)	
Mucinous adenocarcinoma	57 (13.29%)	41 (9.56%)	16 (3.73%)	
Survival status				<0.01
Alive	340 (79.25%)	276 (64.34%)	64 (14.92%)	
Dead	89 (20.75%)	57 (13.29%)	32 (7.46%)	

a p Value between the *low risk* group and *high risk* group.

AJCC, American Joint Committee on Cancer.

### Validation Cohort Demonstrated Stability of the Risk Signature

**FIGURE 7:**
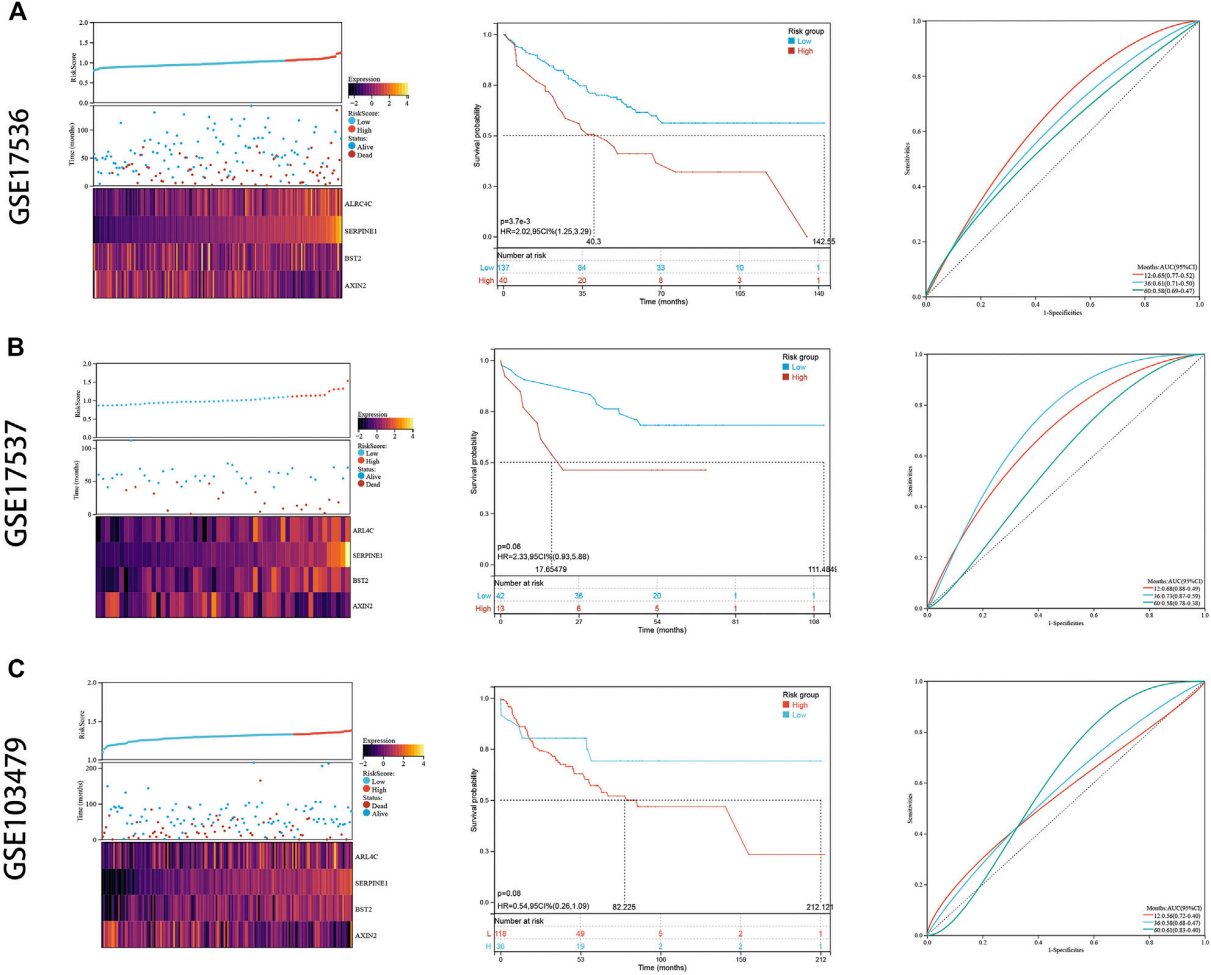
External validation. **(A)** The risk distribution, the Kaplan–Meier survival curve, and the ROC curves in GSE17536. **(B)** The risk distribution, the Kaplan–Meier survival curve, and the ROC curves in GSE17537. **(C)** The risk distribution, the Kaplan–Meier survival curve, and the ROC curves in GSE103479.

To externally validate the prognostic ability of the established risk signature, we calculated risk scores for patients in another three independent cohorts (GSE17536, GSE17537, and GSE103479) using the same formula (Figures 7A–C). Consistently, colon cancer patients with higher risk scores had lower OS rate and shorter OS time in the validation cohorts. The ROC analysis also indicated that the risk signature had a promising prognostic value for patients with colon cancer in the validation cohort (AUC for GSE17536: 1 year = 0.65, 3 years = 0.61, 5 years = 0.58; AUC for GSE17537: 1 year = 0.68, 3 years = 0.73, 5 years = 0.58; AUC for GSE103479: 1 year = 0.56, 3 years = 0.58, 5 years = 0.61). These results showed that the risk signature had an effective and stable OS-predictive ability for colon cancer patients.

### *In Vitro* Validation Demonstrated High Expression of Model Genes in Colon Cancer at Transcriptional and Protein Levels

**FIGURE 8:**
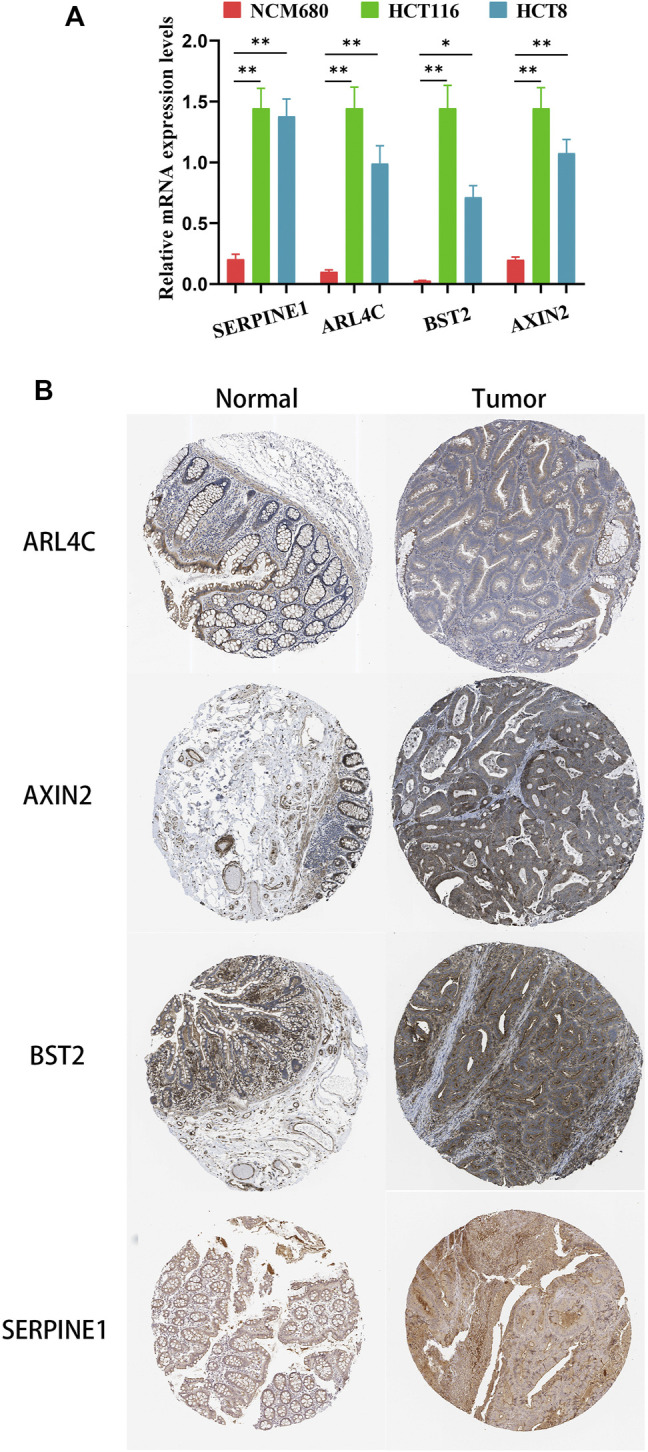
The expression of these four genes in colon cancer cells and tissues. **(A)** The relative mRNA levels of the four genes in normal colon epithelial cells (NCM-460) and colon cancer cells (HCT116, HCT8) by qPCR. **(B)** Representative IHC images of the four genes in colon cancer and normal colon tissues.

To confirm that these four genes are indeed highly expressed in colon cancer, the expressions of these four genes were detected by quantitative PCR (qPCR) in normal colon epithelial cells (NCM-460) and colon cancer cells (HCT116 and HCT8). The results showed that the expression levels of these four genes in colon cancer cells were obviously higher than those in normal colon epithelial cells (Figure 8A). Furthermore, we confirmed the protein expression profiles of these four genes in human tissues. As showed in immunohistochemistry results (Figure 8B), these four proteins were mainly distributed in the cytoplasm or membrane and were upregulated in colon cancer tissues compared with corresponding normal tissues. These results evidently demonstrated that *ARL4C*, *SERPINE1*, *BST2,* and *AXIN2* were upregulated in colon cancer cells and tissues at the transcriptional and protein levels, implying the importance of these four genes in colon cancer pathogens.

### The Risk Signature-based Nomogram Had Better Prediction Ability and Practical Value

Firstly, we used univariate and multivariate Cox analyses to assess whether the established risk signature was an independent prognostic factor for patients with colon cancer (Table 2). Based on the data of colon cancer samples in the TCGA data set, univariate Cox analysis indicated that N stage, M stage, AJCC stage, and risk score were remarkably associated with OS (p < 0.05). Subsequent multivariate Cox analysis further showed that N, M stages, and risk score were independent predictors of OS (p < 0.05). These results indicated that our risk signature, as an independent prognostic indicator, might be useful for clinical prognosis evaluation.

**TABLE 2:** Univariate and multivariate analyses in TCGA-COAD cohort.

Variable	N	Univariate analysis		Multivariate analysis	
		HR (95% CI)	p Value	HR (95% CI)	p Value
Age (years)	429	1.02 (1.00, 1.04)	0.091		
Gender					
Female	202	1 (ref)			
Male	227	1.11 (0.73, 1.70)	0.627		
T stage					
T1	9	1 (ref)			
T2	75	0.48 (0.05, 4.68)	0.531		
T3	297	1.83 (0.25, 13.21)	0.551		
T4	48	6.01 (0.80, 45.04)	0.081		
N stage					
N0	253	1 (ref)		1 (ref)	
N1	99	1.70 (0.98, 2.97)	0.060	0.30 (0.10, 0.87)	0.027
N2	77	4.63 (2.82, 7.58)	<0.001	0.69 (0.25, 1.90)	0.477
M stage					
M0	367	1 (ref)		1 (ref)	
M1	62	4.65 (2.98, 7.24)	<0.001	21.87 (5.21, 91.76)	<0.001
AJCC stage					
Stage I	74	1 (ref)		1 (ref)	
Stage II	170	2.42 (0.72, 8.10)	0.153	2.23 (0.66, 7.50)	0.195
Stage III	123	4.77 (1.45, 15.69)	0.010	9.99 (2.15, 46.42)	0.003
Stage IV	62	13.72 (4.19, 44.94)	<0.001	NA	NA
Risk score	429	7.16 (1.97, 26.00)	0.003	3.12 (0.81, 12.00)	0.011

HR, hazard ratio; CI, confidence interval; AJCC: American Joint Committee on Cancer; NA: not applicable.

**FIGURE 9:**
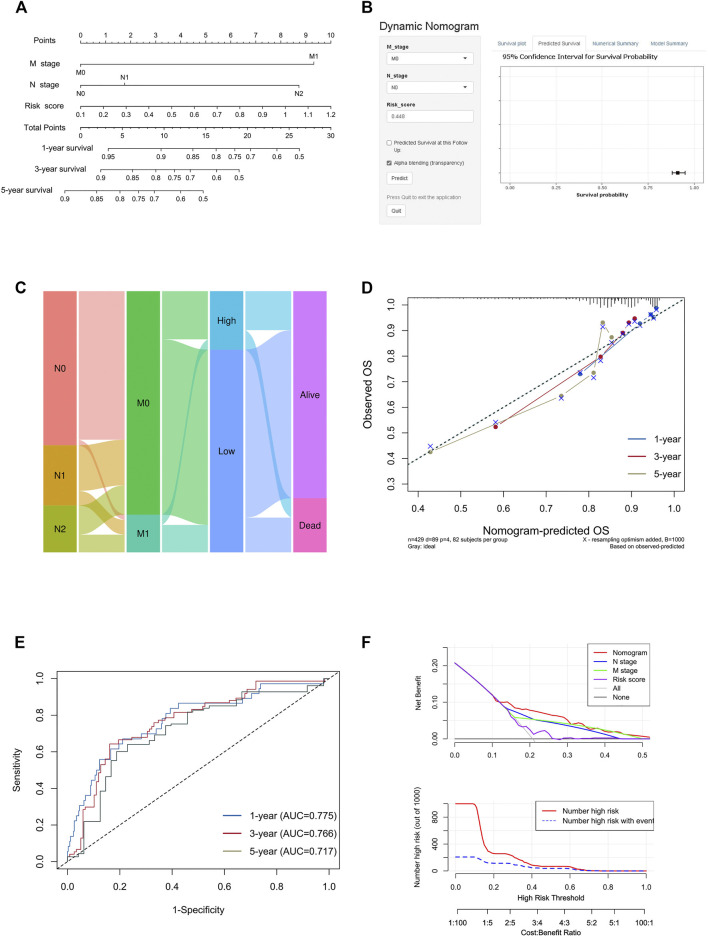
Construction and validation of the nomogram model. **(A)** Construction of the nomogram model combining risk score and prognostic clinicopathological indicators in TCGA-COAD. **(B)** Online dynamic nomogram accessible at https://scxiangya.shinyapps.io/DynNom/, depicting an example for predicting the survival probability of a patient with TxN0M0 colon cancer and a risk score of 0.448. **(C)** Sankey diagram showing the distribution of patients in the *high risk* and *low risk* groups. **(D)** The calibration curve judging nomogram accuracy by comparing the relationship between predicted and observed overall survival. **(E)** Time dependent ROC curves showing the predictive ability of the nomogram. **(F)** Decision curve analysis curves and clinical impact curves evaluating the benefit when using the nomogram model.

To create a clinically applicable quantitative tool to predict the OS of colon cancer patients, we constructed a nomogram model including the risk score, N stage, and M stage in the TCGA data set (Figure 9A), which was available online (https://scxiangya.shinyapps.io/DynNom/) as screenshot in Figure 9B. The Sankey diagram exhibited the distribution of the clinicopathological features of the patients in different groups (Figure 9C). Calibration plots using 1,000 booted resampling revealed perfect concordance regarding the observed vs. predicted rates of 1-, 3- and 5-year OS in the TCGA-COAD cohort (Figure 9D). The ROC analysis also indicated that the nomogram had a stable and robust power in predicting the OS for colon cancer patients (AUC: 1 year = 0.775, 3 years = 0.766, 5 years = 0.717; Figure 9E). The DCA result indicated that the model combining prognosis-related clinicopathologic characteristics and risk signature conferred a better predictive potency than the three-factor model alone (Figure 9F).

## Discussion

Colon cancer is one of the major malignant tumors of the gastrointestinal tract, and approximately 600,000 people die from it every year (Bray et al., 2018; Siegel et al., 2020b), although 5-year survival for colon cancer has approached 65% with improved surgical methods and subsequent treatments in developed countries (Miller et al., 20192019). For colon cancer patients who present with local invasion or distant metastasis, the mortality rate is very high (Misale et al., 2012; Edwards et al., 2014; Fang et al., 2017). Therefore, there is an urgent need to find some new predictive parameters or therapeutic targets that are highly correlated with prognosis. This helps us to establish an early warning system in advance to rapidly identify patients with more critical conditions in clinical work to guide the development of subsequent treatment regimens and the prediction of survival outcomes.

Although it has long been recognized that the immune cells play an important role in tumor initiation and development (Fridman et al., 2012), these insights have not made a major influence on routine clinical practice. Moreover, the transcriptomic correlation of immune infiltration in cancer tissues on diagnosis and prognosis has attracted substantial interest. However, very few of these studies focused on the association between the difference of the immune cell composition and prognosis in colon cancer.

In this study, we first analyzed 28 immune cell types to divide TCGA-COAD into two unique immune patterns: the Immunity_H group and Immunity_L group. The two groups showed significant differences in anti-tumor immune activity, immune cell infiltration, and response to immune checkpoint blockades. Regarding the specific cell types, the complex and diverse immune cells in the TIM include T lymphocytes (70–80%), B lymphocytes (10–20%), macrophages (5–10%), NK cells (<5%), and dendritic cells (1–2%) (Frankel et al., 2017). Additionally, Tregs and TAMs contribute to tumor escape with immune suppressive activity and inhibit anti-tumor responses. Immune cells infiltrating tumors mediate the TIM and thereby influence tumor prognosis (Wu et al., 2013). In this study, we found that effector memory CD4 T cells, activated B cells, eosinophils, and Th17 cells were positively correlated with patient survival prognosis, while the immunosuppressive cells MDSCs were negatively correlated with survival prognosis. Tumor immunotherapy is known to act as a tumor suppressor by acting through these immune cells, so we could screen out colon cancer patients who could benefit from immunotherapy based on different expression levels of immune cells.

It is of great significance to find which immune-related genes that play important roles in the development of the colon cancer and the prognosis of the patient. By specific algorithms, we identified a group of immune-related genes that predict the prognosis of colon cancer patients. With further screening and model construction, four genes including *ARL4C*, *SERPINE1*, *BST2*, and *AXIN2* were singled out to be highly associated with prognosis. It has been found that overexpression of *ARL4C* might contribute to the tumorigenesis and lead to worse prognosis in colorectal cancer (Chen et al., 2016), which supported the result of our study. A recent study has found that *SERPINE1* participated in colon cancer microenvironment remodeling and immune cell infiltration (Wang et al., 2021). This explained why patients with overexpression of *SERPINE1* had poor prognosis in our study. Moreover, the prognostic significance of *BST2* in colon cancer has been put forward as early as 2015 (Chiang et al., 2015). And finally, *AXIN2* has been consistently classified as a tumor suppressor gene in colorectal cancers both *in vivo* and *in vitro* (Church and Fazio, 2005; Waaler et al., 2012). However, it should be noted that *AXIN2* was previously identified as a potent tumor promoter instead of a suppressor, as it was found to exert global control over gene expression networks which were critical for tumor-invasive and metastatic behavior (Wu et al., 2012). Its precise function in the carcinomatous state may require further studies. In general, these four genes are promising prognostic molecules in colon cancer. The constructed model can well distinguish colon cancer patients and predict prognosis, thereby helping to develop individualized treatment options based on survival risk.

The aim of this study is to construct a model composed of prognostic immune related genes, which can robustly predict prognosis. The multivariate Cox regression analysis result showed that the survival time of the *high risk* group was significantly lower than that of the *low risk* group. This shows that our model can be used as an independent prognostic factor for colon cancer patients. According to the nomogram model, the survival rate of colon cancer patients is consistent with the actual situation. This indicates that the model can well distinguish colon cancer patients and outperform clinical parameters alone. Combining the fact that there was a significant difference in the immune cell constitutions between the Immunity_H and Immunity_L groups, we hypothesized that the immune-related genes may affect the tumor prognosis by affecting the immune infiltration. Consequently, the responses rates to immune checkpoint blockade were significantly different between the groups with distinct immune landscapes. It is thus suggested that the poor prognosis of patients in the Immunity_L group may be due to the immunosuppressive microenvironment.

However, there are still some limitations in our study. Due to insufficient clinical information in the three GEO cohorts, the nomogram model failed to be validated, and the AUC values of ROC curves for the risk signature were not high due to the limited sample size. Therefore, we will further validate this prognostic model in other independent large cohorts to ensure the reliability of our model. Moreover, functional experiments are also needed to further reveal the interplay between immune related genes and tumors.

## Conclusion

We identified two distinct immune patterns by analyzing clinical and transcriptomic information from colon cancer patients, which exhibited distinct tumor purity and immune composition. A subsequently constructed immune-related gene-based prognostic model as well as a nomogram model was closely related to the prognosis of colon cancer patients to predict prognosis more precisely, thereby guiding risk stratification and treatment regimen development for colon patients.

## Data Availability

The original contributions presented in the study are included in the article/Supplementary Material, further inquiries can be directed to the corresponding authors.
