# Supplementary material for: Molecular Patterns Based on Immunogenomic Signatures Stratify the Prognosis of Colon Cancer
Source: Front Bioeng Biotechnol. 2022 Feb 14;10:820092. doi: 10.3389/fbioe.2022.820092 (PMC8884696; doi:10.3389/fbioe.2022.820092)
Supplement: Supplementary file 4 [file DataSheet2.docx]

library(DynNom)

library(survival)

library(rms)

library(foreign)

dat=read.csv("clinical_information.csv",header = T)

dat$M_stage=as.factor(dat$M_stage)

dat$N_stage=as.factor(dat$N_stage)

dat$status=ifelse(dat$status=='Alive',0,1)

dat$time=dat$time/30

dd <- datadist(dat)

options(datadist="dd")

f1<- cph(Surv(time, status) ~ M_stage + N_stage+ Risk_score, surv=T, data=dat)

surv <- Survival(f1)

nom <- nomogram(f1, fun=list(function(x) surv(12, x), function(x) surv(36, x),

function(x) surv(60, x)), lp=F, funlabel=c("1-year survival", "3-year survival", "5-year survival"),

maxscale=10, fun.at=c(0.95, 0.9, 0.85, 0.8, 0.75, 0.7, 0.6, 0.5))

plot(nom)

DynNom(f1,dat,covariate = "numeric",clevel = 0.95)

DNbuilder(f1,dat,covariate = "numeric",clevel = 0.95)
